# Tailoring heterogeneities in high-entropy alloys to promote strength–ductility synergy

**DOI:** 10.1038/s41467-019-13311-1

**Published:** 2019-12-09

**Authors:** Evan Ma, Xiaolei Wu

**Affiliations:** 1 Department of Materials Science and Engineering, Johns Hopkins University, Baltimore, MD 21218 USA; 2 State Key Laboratory of Nonlinear Mechanics, Institute of Mechanics, Chinese Academy of Sciences, 100190 Beijing, China

**Keywords:** Mechanical properties, Metals and alloys

## Abstract

Conventional alloys are usually based on a single host metal. Recent high-entropy alloys (HEAs), in contrast, employ multiple principal elements. The strength of HEAs is considerably higher than traditional solid solutions, as the many constituents lead to a rugged energy landscape that increases the resistance to dislocation motion, which can also be retarded by other heterogeneities. The wide variety of nanostructured heterogeneities in HEAs, including those generated on the fly during tensile straining, also offer elevated strain-hardening capability that promotes uniform tensile ductility. Citing recent examples, this review explores the multiple levels of heterogeneities in multi-principal-element alloys that contribute to lattice friction and back stress hardening, as a general strategy towards strength–ductility synergy beyond current benchmark ranges.

## Abstract (web summary)

High entropy alloys are a departure from conventional alloy design. In this review, the authors examine how combinations of hetererogeneities specific to high entropy alloys can overcome the strength–ductility trade-off inherent to traditional alloys.

## Introduction

Metals and alloys are essential materials for manufacturing and load-bearing applications. For such structural materials, strength and ductility at room temperature (RT) are two baseline mechanical properties. The yield strength, *σ*_y_, is the stress at which a material begins to deform plastically; it represents the upper limit to stresses which can be applied to a material without a macroscopic permanent shape change. Ductility, on the other hand, is a measure of a material’s ability to deform plastically after yielding before fracture, usually expressed as percent elongation to failure (*ε*_f_) in a uniaxial tensile test. The portion of the ductility before plastic instability^1^, *ε*_u_, is the uniform plastic strain that can be sustained before the onset of localized deformation (such as necking or shear banding); a large *ε*_u_ is especially important in metalworking to ensure uniform shaping. A hallmark of metals is their great ductility, together with tunable strength that can be readily elevated through well-established processing routes.

**Fig. 1:**
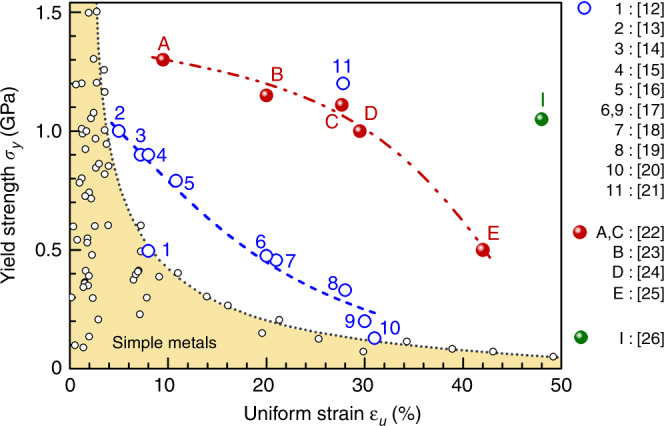
Yield strength versus uniform tensile strain. The yellow-shaded area under the banana-shaped dotted curve covers the strength–ductility data of typical conventional metals. The reader is referred to the literature^11^ for the data points already summarized previously. The open blue circles along the blue dashed line (a guide to the eye) are HNM examples, from refs. ^12–21^, examples of recent heterogenous HEAs are shown along the red dashed line, see solid red circles A–E from refs. ^22–25^ Case I, the solid green circle, is for a very recent complex alloy containing multicomponent intermetallic nanoparticles^26^.

In general, the goal is to raise the yield strength (*σ*_y_) as much as we can, while conceding as little ductility as possible and preserving a high *ε*_u_. This is challenging because strength and ductility usually exhibit a trade-off: a gain in *σ*_y_ is normally accompanied by a sacrifice in *ε*_u_^2–11^. This is shown in the shaded banana-shaped region in Fig. 1 for simple metals. These (mostly elemental) metals, in their unstrengthened form (i.e., coarse-grained and annealed state with no added solutes and precipitates), have relatively low strength and high ductility. When *σ*_y_ approaches gigapascal level through strengthening routes such as cold working, solid solution hardening, and grain refinement, there is a fast loss of uniform tensile strain: *ε*_u_ decreases drastically to less than a few percent (Fig. 1). In a previous review^11^, we presented a perspective that heterogeneities intentionally introduced into a metal, such as a grain/twin size distribution/gradient, lamellae, and hierarchical defects derived thereof, promote strain hardening and hence uniform tensile ductility, leading to noticeably improved strength–ductility combinations (Fig. 1). This design strategy of heterogeneous nanostructured metals (HNMs)^11^ serves as the starting point of the present review.

We note that in Fig. 1 even the strength–ductility improvement achieved for HNMs along the blue dashed line^12–20^ remains a trade-off, albeit at a level higher than conventional metals (yellow-shaded area), leaving much room in the strength–ductility space to expand into. The data points along the blue dashed line all share one feature in common: they have significant nanoscale heterogeneities in their microstructure^11^. This suggests that purposely increased heterogeneity that promotes nonhomogeneous plastic deformation is essential to push the strength–ductility envelope. This is not surprising, as many heterogeneous materials such as precipitation-hardened alloys, multiphase alloys, and composites, have been developed to possess high strength while retaining good ductility. Nevertheless, when limited to a single base metal, the potential for property enhancements has been exhausted; indeed, the gain in (*σ*_y_, *ε*_u_) along the blue dashed line over conventional metals is rather limited. One therefore naturally wonders if concentrated alloys can offer (*σ*_y_, *ε*_u_) beyond current benchmark ranges^12–21^. This proposition brings us to the so-called high-entropy alloys (HEAs), also called multi-principal component alloys or complex-concentrated alloys^22–26^. For a systematic introduction to this new paradigm recently emerged in metallurgy, the readers are referred to several review articles^27–31^. HEAs, and their variants such as medium-entropy alloys (MEAs)^32^, started with single-phase alloys (solid solutions) of nearly equiatomic compositions. More recent HEAs have expanded their scope to include compositions further away from the center of the multicomponent phase diagram and alloys with dual or multiple phases^26,33–35^. We highlight several examples in Fig. 1 (data points A through E (refs. ^22–25^) and I^26^). These HEAs^30^ offer high *σ*_y_ and *ε*_u_ that are clearly superior to others. In this review, we will survey the wide range of strength–ductility reported so far for HEAs, focusing on improved strength–ductility synergy through alloy designs that exploit both multiple principal elements and heterogeneities.

### Heterogeneities in HEAs

**Fig. 2:**
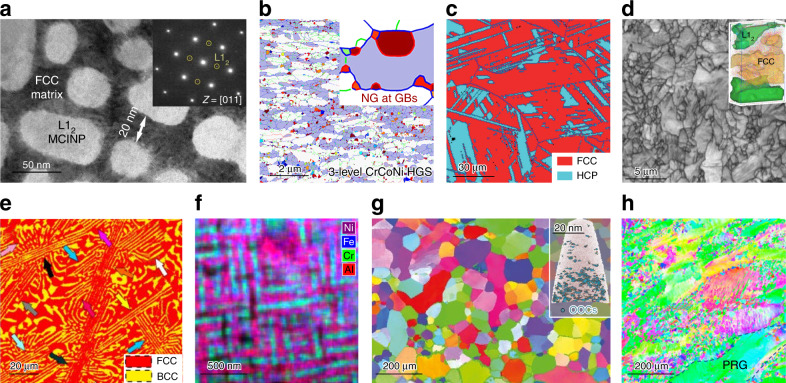
Example microstructures of highly heterogeneous HEAs. **a** Multicomponent intermetallic nanoparticles (MCINP)-strengthened FCC (FeCoNi)_86_-Al_7_Ti_7_ (Ll_2_) HEA (from ref. ^26^. Reprinted with permission from AAAS). **b** Heterogeneous grain structured (HGS) CrCoNi MEA^23^. **c** Transformation-induced plasticity (TRIP) dual-phase Fe_50_Mn_30_Co_10_Cr_10_ HEA^33^. **d** Al_0.5_Cr_0.9_FeNi_2.5_V_0.2_ HEA with ordered Ll_2_ precipitates and disordered FCC matrix^38^. **e** Dual-phase eutectic lamellae AlCoCrFeNi_2.1_ HEA^36^. **f** Al_1.3_CoCrCuFeNi HEA of Al–Ni-enriched matrix with Cr–Fe-enriched plates^37^. **g** BCC single-phase TiZrHfNb HEA. Inset: (TiZrHfNb)_98_O_2_ HEA. OOCs ordered oxygen complexes^22^. **h** Hierarchical features infused HGS HEA, consisting of partially recrystallized grains of large size and completely recrystallized grains of fine size ^39^.

Figure 2 highlights new schemes of nanostructured heterogeneities^22,23,26,33,36–39^. Our intent here is to call the readers’ attention to the multilevel heterogeneities enabled by HEAs, and explain how they influence dislocation motion to elevate *σ*_y_ to the gigapascal level, while boosting strain hardening and retaining *ε*_u_ that rivals simple metals.

To set the stage for our discussion, we first define what we mean by “heterogeneities” and why HEAs expanded their scope. We then discuss the roles played by each of the following levels of heterogeneity (although not necessarily in the particular order below), in terms of their individual and synergistic contributions to strength and ductility. In HEAs, the first level of heterogeneity comes from the multiple constituent species in a concentrated solution at the atomic-level. There can be appreciable inhomogeneity, statistically fluctuating from one location to another, in compositional and packing arrangements of the various elements, not to mention local chemical order (LCO) that may develop in the short-to-medium range (nearest neighbors and the next couple of shells). The next level includes tiny and closely spaced clusters and complexes, and precursors of precipitates, that may emerge on the nanometer scale before a second phase becomes identifiable in the nominal single-phase solution. The third level rises from multiphase nanostructures that evolve out of the parent solution, transforming (part of) the alloy into dual-phase (DP), precipitation hardened, eutectic lamellae structure, martensites, etc. A fourth level of heterogeneity arises from the defects embedded in the crystal lattice, including nanotwins, stacking faults, and grain boundaries, which can refine the grains along with plastic deformation. Finally, the grain-size distribution may be intentionally made multimodal or graded (e.g., via partial recrystallization) with length scales from a few nanometers up to the micrometer level. These five levels of heterogeneities, both chemical and structural, can now be intertwined in a given HEA due to the complex interaction of multiple principal elements. This constitutes a “hierarchy of heterogeneity” in the microstructure (see examples in Fig. 2), making HEAs unusually prone to nonuniform plastic deformation even when loaded under uniform stresses.

As a starter to incentivize the heterogeneous HEA route to strength–ductility synergy, we first briefly mention one very recent case, the impressive data point I^26^ in Fig. 1. This multicomponent alloy is composed of a large volume fraction (up to 55%) of ductile multicomponent intermetallic nanoparticles (MCINPs), see Fig. 2a. The MCINPs are coherent with, and uniformly distributed in, the face-centered cubic (FCC) FoCoNi matrix, and have an L1_2_ crystal structure with a complex composition of (Ni_43.3_Co_23.7_Fe_8_)_3_(Ti_14.4_Al_8.6_Fe_2_). Interestingly, this carefully crafted alloy achieves *ε*_u_ ~50%, i.e., the alloy remains as ductile as any unstrengthened elemental metal, but at a *σ*_y_ orders of magnitude higher (well above 1 GPa). This latest case nicely illustrates that complex concentrated HEAs can offer unprecedented strength–ductility well into previously inaccessible territory in Fig. 1.

**Fig. 3:**
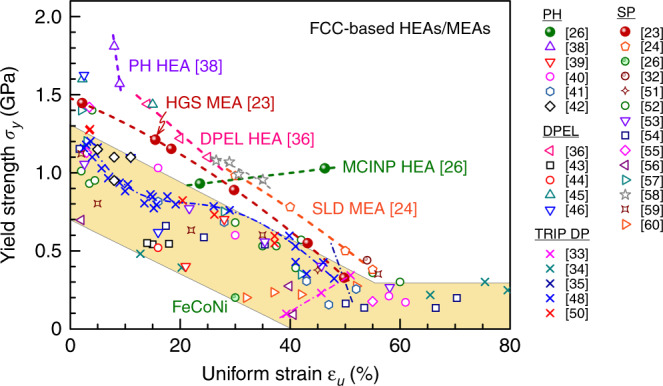
Yield strength versus uniform tensile strain in FCC-based HEAs. Four types of microstructures are summarized in the plot. A few high-performance examples above the yellow band are marked with acronyms and references. Precipitation hardened (PH): two examples are highlighted using green solid circles^26^ and open violet triangles^38^, while the rest cases are shown with open symbols. Dual-phase eutectic structure: a representative of this group is the dual-phase eutectic lamellae (DPEL) in ref. ^36^. TRIP metastable dual-phase (DP): all × symbols, e.g., ref. ^33^. Single-phase (SP) FCC: highlighted in red solid and open circles are the HGS-CoCrNi MEA^23^ and the VCoNi MEA with severe lattice distortion (SLD)^24^, respectively; the rest in this SP group is shown with open symbols.

In the following sections, we shall summarize (*σ*_y_, *ε*_u_) of multi-principal-element alloys. We first discuss FCC-based HEAs in Fig. 3, followed by body-centered cubic (BCC)-based HEAs, and finally advanced steels based on twinning-induced plasticity (TWIP) and transformation-induced plasticity (TRIP) effects. The majority of the FCC and BCC groups are single-phase solid solutions (although many recent cases have involved additional hardening from precipitates and a second phase), while the third group are (metastable) DP alloys relying heavily on hardening induced by a martensitic transformation. We showcase the range of tensile properties that have been achieved recently and highlight that tweaking heterogeneous microstructures can be quite powerful in lifting the strength–ductility balance.

### Strength–ductility of FCC-based HEAs

Figure 3 shows the data for FCC-based HEAs based on refs. ^23–26,33–60.^ While overall still a trade-off between *σ*_y_ and *ε*_u_, the (*σ*_y_, *ε*_u_) combinations are nonetheless superior to previous single-phase HNMs (when compared with the blue dashed line in Fig. 1). Some recently developed good performers are highlighted in Fig. 3, where the strength–ductility properties are often due to intentionally introduced heterogeneities such as precipitates^61^, which is where we shall begin. Note that the metastable nature of the HEAs as supersaturated solid solutions, from which phase decomposition produces hardening (intermetallic) precipitates, together with mechanically driven transformations into dual/multiphase microstructures, has recently been reviewed^31,47^.

Some of the FCC HEAs in Fig. 3 contain heterogeneities by way of precipitation hardening (PH)^26,38–42^, such as Ni_3_(Al, Ti)-type precipitates in FCC-CoCrFeNi HEA^40^, and *σ* and *μ* particles in CoCrFeNiMo_0.3_ (~7 at% Mo)^41^. The yield strength has been tuned to reach 816 MPa with a total elongation of ~19%^41^. In this strategy, the precipitates interact with dislocations to provide both strength and increased capability of strain hardening, in a similar way to precipitation hardened Al alloys^62,63^. The intermetallic particles, although hard and brittle, do not cause fracture because the toughening of the surrounding FCC HEA matrix that has very good ductility. For an extreme PH case with high strength, a recent work^38^ via spinodal decomposition in Al_0.5_Cr_0.9_FeNi_2.5_V_0.2_ reached as high as 50 vol% of Ni_3_Al-type L1_2_-structured as well as 6% of BCC structured precipitates in FCC matrix, boosting the yield strength to ~1.8 GPa at a ductility of 9% (see Fig. 2d and the PH line in Fig. 3). Another multiphase heterogeneity design is the dual-phase eutectic lamella (DPEL) HEA^36,43–46^. One example is a eutectic HEA, which in addition to the FCC phase contains a second BCC phase as well as Cr-enriched nanoprecipitates^44^. Most recently, a heterogeneous DP (FCC + B2) lamella HEA, inheriting the as-cast eutectic microstructure seen in Fig. 2e, was also able to produce a good strength–ductility balance of 1.4 GPa and 14% elongation^36^ (see the DPEL line in Fig. 3).

In Fig. 3 we also include the metastable DP Fe_80−*x*_Mn_*x*_Co_10_Cr_10_ system^33–35^, because it is based on an FCC HEA. This group has purposely enhanced metastability due to lowered stacking fault energy (SFE), by tuning the Mn content towards Fe_50_Mn_30_Co_10_Cr_10_. This alloy, seen in Fig. 2c, starts out heterogeneous: in this case it contains partial martensitic transformation to a hexagonal close-packed (HCP) phase, induced thermally upon cooling from the high-temperature single-phase FCC region. The phase boundaries contribute additional obstacles to dislocation slip, adding interphase strengthening to intraphase dislocation strengthening. Moreover, upon loading the FCC phase with low mechanical stability also undergoes deformation-stimulated martensitic transformation, facilitated by the pre-existing HCP plates as nuclei. The displacive transformation to HCP provides strain hardening dynamically during tensile straining, supplementing dislocation storage to sustain a large uniform elongation. This TRIP-assisted DP alloy (further discussed below) claims to overcome the *ε*_u_ – *σ*_y_ trade-off^33^: *ε*_u_ and *σ*_y_ rise at the same time (see the pink dash-dotted line in Fig. 3). One drawback is that this TRIP-assisted DP alloy has a relatively low *σ*_y_. If these alloys are hardened before tensile testing to raise *σ*_y_ to the GPa level using cold work or small grain size, the strain-hardening rate after yielding becomes inadequate to sustain large *ε*_u_. In other words, the trade-off comes back at high *σ*_y_ levels (see the blue dash-dotted line^48^ in Fig. 3).

An additional aspect of these phase-transformation microstructures is that the SFE can be tuned via alloy composition. For example, it has been shown that enriching Co (up to 45%) in CoCrFeNi encourages twinning and martensitic transformation^49^. Also, with a near-zero but positive SFE, the transformation can go both ways and becomes bidirectional^49,50^. Such multiple forward and reverse deformation-driven martensitic transformation events can refine the alloy to a nanolaminate structure and provide extensive work-hardening capacity and material strengthening while retaining good ductility^49^.

Again, the best property combination appears to be the latest case I in Fig. 1, the FCC-MCINP alloy^26^ (see green solid ball in Fig. 3). The unprecedented *ε*_u_ ~50% at *σ*_y_ above GPa, are attributed to multistage work hardening^26^, with pronounced activities of dislocations interacting with the uniformly distributed high-density MCINP (30–50 nm, in both their size and separation distance, see Fig. 2a). In addition to these second-phase heterogeneities, there are also heterogeneities induced by deformation. Directional dislocation substructures such as dense dislocation arrays and walls create long-range back stresses, contributing to strain hardening together with the large increase of short-range effective stresses from accumulated forest dislocations. Microbands similar to low-angle grain boundaries are also induced by deformation. This alloy’s strength and ductility increase simultaneously from that of its counterpart FeCoNi base alloy without the nanoparticles (labeled in Fig. 3 for comparison). Such a simultaneous increase in strength and ductility with respect to the baseline alloy was known to be feasible previously, e.g., by adding and controlling nanoprecipitate features^62,63^. But note that even with reference to unstrengthened elemental metals, the data point I (Fig. 1) is unique in that it shows no strength–ductility trade-off: i.e., strengthening to GPa strength with no sacrifice of *ε*_u_ at all.

**Fig. 4:**
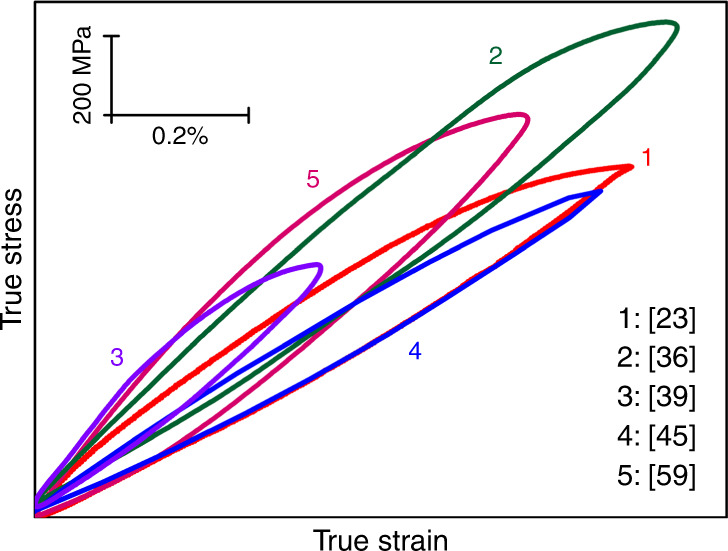
Hysteresis loop in load–unload–reload tensile stress–strain curve of HEAs/MEAs tested at RT. These loops are recorded at respective maximum uniform tensile strain, i.e., at a strain close to necking on the stress–strain curve. The loop widths cited here are by far the widest for all alloys reported in the literature to date. This indicates that the heterogeneous microstructures in these HEAs generate high back stresses that contribute to strengthening and strain hardening. The back stresses can be estimated from the loop using unload residual plastic strain and yield stresses upon unloading and reloading^15,23^. After the first report in ref. ^23^, similarly wide hysteresis loops have been observed in other heterogeneous HEAs, as summarized in this plot.

**Fig. 5:**
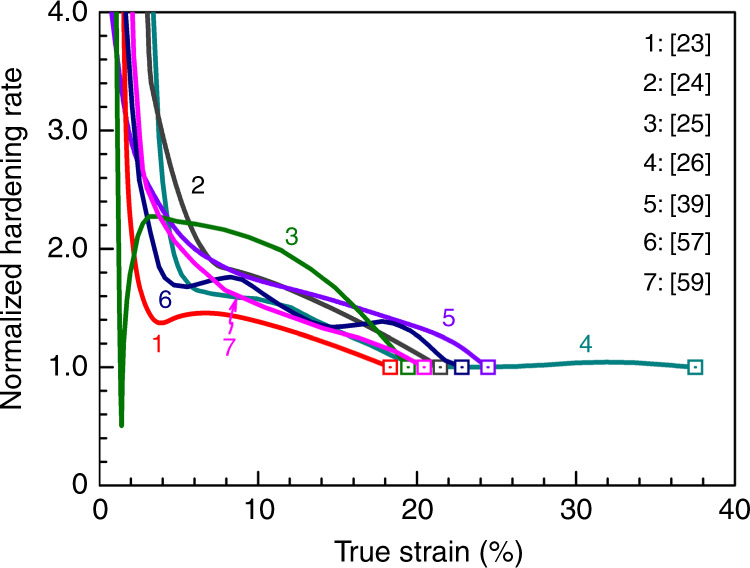
Strain-hardening responses. Normalized strain-hardening rate (${\mathrm{\Theta }}\,=\,\frac{1}{\sigma } \cdot \frac{{\partial \sigma }}{{\partial \varepsilon }}$) versus true tensile strains in FCC HEAs. All alloys showed GPa-level yield strength. The Considère plastic instability point (when ${\mathrm{\Theta }}$ descends to unity, marked using squares) is delayed to large tensile strain.

A question naturally rises as to what kind of strength–ductility balance can be achieved in single-phase FCC solutions without precipitates or a second phase. Can one achieve (*σ*_y_, *ε*_u_) similar to PH cases, in the absence of a second phase? To address this, in Fig. 3 we also highlight FCC alloys^23,24,32,51–60^ heterogeneously structured but without second phases. One example is a single-phase CrCoNi MEA (red solid balls^23^) with abundant heterogeneities such as a purposely designed heterogeneous grain structure (HGS) (Fig. 2b^23^). Figure 3 shows that this HGS can deliver a strength–ductility combination comparable with the precipitation-hardened FCC cases. This HGS-MEA is also notable because heterogeneities are introduced both through sample processing and dynamically during tensile straining. To achieve both high *σ*_y_ and large *ε*_u_^23^, the alloy first has an HGS that spans the nano-to-micro grain-size range to reach a gigapascal yield strength. This ultrafine heterogeneity is produced via partial recrystallization, taking advantage of the low SFE of this MEA, which facilitates the generation of nanoscale corner twins in recrystallization. Second, plastic deformation is nonhomogeneous after yielding. The elasto–plastic transition through load transfer and strain partitioning among grains of different sizes leads to an upturn of the strain-hardening rate. Third, dynamic recrystallization continues at RT upon tensile straining, generating more corner twins which evolve into nanograins with high-angle grain boundaries. Fourth, an increasing number of deformation nanotwins and faults via partial dislocations are embedded into the grains on the fly, rather than just full dislocations that run across the grains and annihilate easily. In particular, an unusually wide hysteresis loop in load–unload–reload stress–strain curve (see Fig. 4) demonstrates that the nonhomogeneous plastic deformation in the HGS imparts an additional long-range hardening effect from unusually high back stresses. After this example, several HEAs/MEAs have also been shown to exhibit wide hysteresis loops^36,39,45,59^ (see Fig. 4). With increasing tensile strain, these loops widen, and the back stresses derived from these loops increase, contributing to strain hardening. The HGS is therefore dynamically refined and reinforced, sustaining a high strain-hardening rate to large tensile strains^23^ (see Fig. 5), allowing to achieve a *ε*_u_ in excess of 20% despite its gigapascal *σ*_y_. A similar strategy of partial recrystallization with bimodal grain sizes was also used in an FCC VCrMnFeCoNi HEA^25^, FCC Al_0.1_CoCrFeNi HEA^57^, Cr_20_Fe_6_Co_34_Ni_34_Mo_6_^58^, and another CrCoNi MEA^59^; all of which take advantage of the ensuing dynamic recrystallization. Additional hierarchical heterogeneities, including B2 precipitates, are sometimes also introduced to help elevate the back stresses^39^. Dynamic deformation twinning is akin to TWIP and similar to dynamic phase transformations that generate heterogeneities (TRIP)^33^.

To further elevate the yield strength of FCC HEAs/MEAs, constituent elements can be selected to intentionally increase the atomic size mismatch to cause more lattice distortion, such that the lattice friction to dislocation motion, i.e., the Peierls stress, is maximized. To this end, a recently developed VCoNi MEA^24^, although still a single FCC phase, achieved near-GPa *σ*_y_ together with *ε*_u_ ~30% until *ε*_f_ ~38% (SLD-dashed line in Fig. 3). The authors report that this MEA has the highest friction stress so far for FCC HEAs/MEAs as well as a high sensitivity to grain boundary strengthening.

### Strength–ductility of BCC-based HEAs

**Fig. 6:**
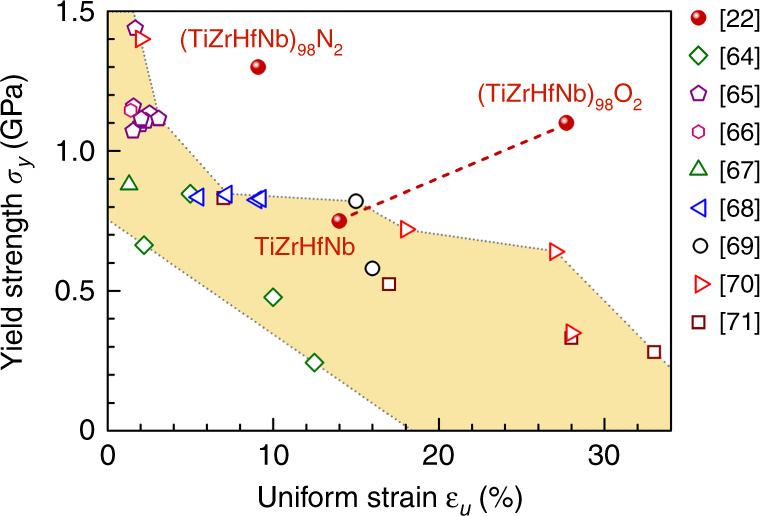
Yield strength versus uniform tensile strain observed in BCC HEAs. Note that heterogeneity introduced by the addition of 2% oxygen solutes into BCC-TiZrHfNb HEA (solid red circle) markedly promotes its strength–ductility synergy (dashed line)^22^.

Figure 6 summarizes the data for the BCC-structured HEAs/MEAs^22,64–72^. We observe a strength–ductility trade-off band similar to the yellow one for FCC HEAs in Fig. 3. BCC HEA data is limited thus far, but recent interest has been kindled by refractory HEAs, which are single-phase BCC solid solutions composed of various refractory metals such as titanium, zirconium, hafnium, vanadium, niobium, tantalum, molybdenum, and tungsten^30,64,72^. Research into these refractory HEAs is motivated by their potential to rival Ni-based superalloys in terms of high strength at elevated temperatures. At RT, refractory HEAs exhibit a *σ*_y_ up to the level of 2 GPa^72^, but most of them show some ductility only in compression. The lack of RT ductility is related to a drastic rise of the ductile-to-brittle transition temperature, at which the fracture stress and the yield stress cross^72^, when concentrated alloying elements are added into a base BCC metal. The exact mechanism is yet to be fully understood. The refractory BCC HEAs that show at least some tensile ductility are compared in Fig. 6, which are mostly based on the Ti–Zr–Hf–Nb–Ta system: the as-cast BCC equiatomic TiZrHfNbTa alloy shows a *σ*_*y*_ of ~830 MPa^65,66^, which can be further elevated via subsequent cold rolling and annealing^68^, together with a tensile elongation to failure of ∼9%. BCC-MEAs with tensile ductility have also been reported recently^64^, and are included in Fig. 6.

Similar to FCC-based HEAs, adding heterogeneities into BCC-based HEAs can be an effective way to boost strength and strain hardening simultaneously. Precipitates have rarely been used in these BCC HEAs thus far, perhaps because the matrix is of low ductility to begin with. Here we highlight a recent case^22^ where the authors utilized precipitate precursors instead. They formed nanoscale clusters by adding oxygen solutes into the base BCC-TiZrHfNb HEA. These solute atoms, kept at a low concentration of ~2 at% such that oxygen embrittlement does not take over, interact with Ti and Zr preferentially, and segregate to form a high density of nanoscale (O, Ti, Zr)-complexes that are a couple of nanometers in size and a few nanometers apart, see inset of Fig. 2g. These widespread local complexes interact with moving dislocations, elevating strength while simultaneously enhancing strain hardening and ductility^22^ (see the dashed line in Fig. 6, where this (TiZrHfNb)_98_O_2_ HEA stands out). The authors attribute this to the pinning of dislocations by oxygen complexes, and homogenization of strains via wavy slip of dislocations that facilitate cross-slip and dislocation multiplication, rather than planar slip that localizes strains. It should be noted that while^22^ claimed to overcome the strength–ductility trade-off, the comparison was to an already-strengthened state with reduced ductility: the as-cast TiZrNbHf in Fig. 6 was solution hardened by multiple elements to *σ*_y_ > 750 MPa^22^, with ductility already reduced to ~14%. The ductility increase to ~28% due to oxygen complexes therefore only restores tensile elongation approaching unstrengthened BCC metals. Also note that this ductility is not truly uniform strain per se, but a nominal *ε*_u_ thanks to very diffuse necking after violation of the Considère criterion for instability^73^ (see the true stress data in ref. ^22^). This is different from normal BCC elemental metals, but not uncommon for high-strength BCC alloys. Similar to FCC-based HEAs, deformation-induced phase transformations can also be induced in BCC-based HEAs, which will be discussed in the next section.

As for HCP-HEAs, they are still rare at present. Due to limited slip systems, they do not seem appealing for ductility. But opportunities may also emerge because HEAs offer the flexibility to tune alloy composition such that the c/a ratio can be adjusted to facilitate mechanisms such as <c + a> slip to mediate ductility. A very recent example along this line is ref. ^71^

### Strength–ductility promoted by phase transformation

**Fig. 7:**
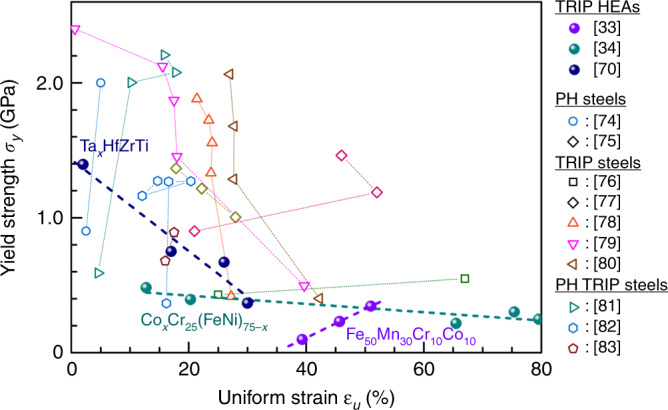
Yield strength versus uniform tensile strain observed in recently reported multi-principal-element steels. These advanced steels use TRIP and TWIP effects to boost strain hardening.

In Fig. 7 we summarize some recently designed HEAs that emphasize the TRIP effects^33,34,69,74–83^; some of these have already been included in Fig. 3, e.g., ref. ^33^, and in Fig. 6, e.g., ref. ^69^. Stress-assisted martensitic transformation creates a high volume fraction (65%) of an internally twinned martensite phase in BCC-Ti_35_Zr_27.5_Hf_27.5_Nb_5_Ta_5_ HEA^69^. In ref. ^69^, the authors deliberately stay away from the equiatomic stable BCC composition, such that the martensitic transformation and associated TRIP effect kick in during tensile deformation, leading to a twofold increase in the strain-hardening rate that sustains *ε*_u_ to 17%. In ref. ^70^, the authors compositionally adjust the metastable TaHfZrTi BCC HEA by reducing the Ta content^70^, leading to thermally and mechanically induced HCP martensite. This helps sustain strain hardening and consequently good combinations of strength–ductility.

Here in Fig. 7, we include many more steel cases as HEAs exhibiting the TRIP effect (where the martensitic transformation is taken advantage of during tensile deformation to elevate strain hardening and sustain *ε*_u_). Many such advanced steels possess good combinations of strength and ductility (see the ten examples in Fig. 7). The HEA modification to known TRIP steels highlights the metastable nature of HEAs, where phase transformations can often be driven by stresses during tensile straining. The evolving two-phase microstructure adds resistance to interphase dislocation slip, promoting strength. Simultaneously, the in situ phase transformation during straining is a potent mechanism for strain hardening, as mentioned in earlier sections.

To recapitulate, the three groups of HEAs surveyed in the three sections above cover a wide strength–ductility range, situated, as expected, above previous HNMs based on a single host metal. Heterogeneities are abundant, at various levels from defects stored in the crystals to multimodal grain sizes to multiphase nanostructures. In the following sections, we will discuss, from a more mechanistic perspective, how the heterogeneities act in concert to strengthen as well as to promote strain hardening (and therefore uniform ductility) of these HEAs.

### Mechanisms behind elevated strength

We begin by first looking at yield strength and the key factors that elevate the strength of HEAs. Of course, the traditional plethora of metallurgical tricks such as grain refinement and cold work, even PH, remain powerful routes; these have been applied to HEAs to achieve improved properties, as summarized above. Here we single out physical mechanisms unique to solutions with high concentrations of constituent elements, which are not present in conventional solution strengthening in dilute alloys with noninteracting solute atoms.

**Fig. 8:**
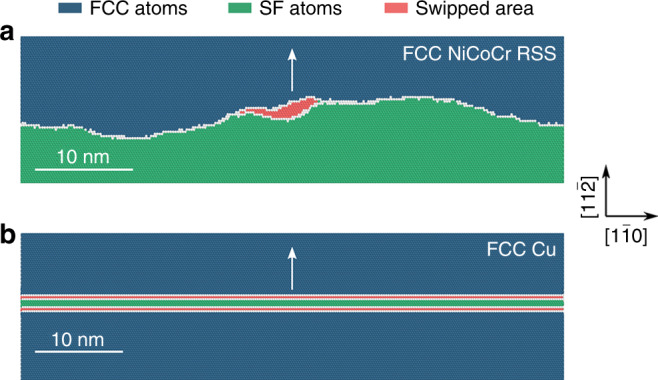
Dislocation motion in FCC lattice. The molecular dynamics simulation was performed using embedded atom method (EAM) potentials, see ref. ^89^ for details. To observe configurations associated with the intrinsic potential valley, all configurations were cooled to zero temperature and unloaded to zero stress from stressed MD simulations at 300 K, and further relaxed by energy minimization. **a** Wavy dislocation line in FCC-NiCoCr MEA random solid solution (RSS). The leading partial (LP) dislocation moves forward in the direction marked with an arrow, one local segment at a time, while the trailing partial (TP) lags far behind (out of the picture) because the energy cost associated with erasing the stacking fault (green) is relatively high^89^. The locations of the dislocation line at two times, *t*_1_ and *t*_2_, clearly reveals the nanoscale segment detrapping mechanism: the swept area (scaling with activation volume) in one stick-slip event is shown in red. This is in sharp contrast with the familiar behavior of dislocation in normal FCC, shown in **b** using Cu as example, where a long dislocation line (both the straight LP and TP shown in the figure) marches easily as a whole from one Peierls valley to the next.

The advent of HEAs accentuates the need to understand why the *σ*_y_ of multi-principal element solutions is intrinsically higher than simple metals or dilute solutions (at the same grain size and percent cold work). One could view HEAs as a “cocktail” solution where the solvent and solutes are no longer clearly definable, pushing solution hardening to an extreme. Strengthening, i.e., increased stress required to move a dislocation, arises from the totality of the interaction energies between the constituents and an individual dislocation^84^. A major contribution to this interaction energy is the elastic interaction of the dislocation stress field with the misfit strain tensor of the solute atom^85,86^. As shown in the model of Varvenne et al.^84^, together with the effective shear modulus, the quantity of misfit volume (which can be viewed as a measure of the lattice distortion^24,87^, the atomic-level pressure, and electron charge transfer^88^) scales with the friction stress. Here we emphasize that the statistical distribution of multiple principal elements in the lattice gives rise to obvious local fluctuations in their concentrations, sometimes with associated variations of LCO and local misfit. The dislocation is attracted to energetically favorable fluctuations and repelled by energetically unfavorable fluctuations. So the dislocation line adopts a waviness, and there is a barrier corresponding to energy cost of moving from a favorable to an unfavorable potential energy fluctuation, together with an elastic bowing energy due to line tension. This collective concentration/structural inhomogeneity raises the energy barrier controlling the stress needed for dislocation moving in the lattice. This behavior is shown in Fig. 8a, obtained using molecular dynamics simulations for a random FCC-NiCoCr MEA^89^. Attempts have also been made recently to observe the effects of heterogeneities in microscopy experiments^90^. In comparison, for the familiar FCC Cu in Fig. 8b as a reference, as expected from known behavior in FCC we observe a long and straight dislocation line that moves easily as a whole from one Peierls valley to the next, as the barriers in between them are very low.

Again, a long straight dislocation in an HEA will reduce its total potential energy by adopting a wavy line, along which some segments reside in regions of favorable compositional fluctuations (Fig. 8a). This is fundamentally different from a pure metal or dilute solutions (Fig. 8b), where the local environments of all the segments of a dislocation line are equivalent; in an HEA all the nanoscale segments behave differently. In addition, there will also be spatially variable SFE even in any given HEA. The “local SFE” and consequently variable dislocation core structure was first reported by Smith et al., for the FCC-CrMnFeCoNi HEA^91^, and the variable partial dislocation dissociation distance was recently shown again using TEM imaging^92^. Moreover, for samples aged at an elevated temperature, e.g., homogenization at 1200 K, LCO can develop to various degrees, from short-to-medium range all the way to ordered domains on nanometer scale^89,93^. The dislocation lines become wavier due to such complex spatial heterogeneities, and the critical resolved shear stress rises with the LCO. Note that unless an HEA is rapidly quenched to RT from a very high temperature (e.g., 2000 K), truly random solutions are rare and LCOs will develop to some extent. This local chemical complexity makes two contributions to additional strengthening. First, LCO heightens spatially heterogeneous SFE and instigates local antiphase boundary energy, resulting in extra restoring forces on a dislocation that is moving to break/randomize the LCOs. The stronger the LCO in a local region, the larger restoring force a dislocation feels on average. Second, the spatially varying LCO promotes nanoscale heterogeneities acting as roadblocks to trap moving dislocations (see Fig. 8a), similar to well-known G-P zones in precipitation-hardened alloys.

Therefore, the dislocation has to navigate through a choppy sea of heterogeneities in an HEA, even if it is nominally a random solution or has only partial chemical order. The mechanism for dislocation movement becomes intermittent “nanoscale segment detrapping”^89^, see Fig. 8a. which entails an activation volume much smaller than in conventional FCC metals. The associated activation energy barrier results in an unusually high lattice resistance to dislocations.

In addition to this lattice friction, heterogeneities on various microstructural levels can be built either during alloy processing or accumulated in situ during tensile straining, further resisting dislocations. In what follows, we mention these different levels briefly, to echo the mechanisms discussed in earlier sections. The strengthening due to dislocation obstacles, such as grain boundaries, dislocation tangles, and second-phase precipitates, is familiar to the metallurgy community. What is special about HEAs is their extraordinary propensity to reach high densities of such dislocation roadblocks. The cases in Fig. 2, showing GPa-level yield strength (Fig. 1), exemplify this via different routes to heterogeneous microstructure. For example, grain size can be made multimodal to span a wide range^23^; hierarchical defects are readily built into the microstructure, e.g., ref. ^39,58^; precipitates and locally segregated/ordered complexes can be induced on nanometer scale^22,61^, and second-phase nanoparticles can be deployed at high numbers^26^, not to mention second phase formed via martensitic transformation^33^. These abundant possibilities all exert additional resistance to cause moving dislocations to stall or pile up. It is then unsurprising that a large fraction of HEAs/MEAs reach GPa-level yield strength and high back stresses.

### Mechanisms facilitating strain hardening and ductility

We now examine the strain-hardening ability in HEAs. We first reiterate that a high strain-hardening rate is key to evading the strength–ductility trade-off. As an example, we know from the Considère criterion^73^ that the necking instability instigating incipient failure sets in when the normalized strain-hardening rate ${\mathrm{\Theta }}\,=\,\frac{1}{\sigma } \cdot \frac{{\partial \sigma }}{{\partial \varepsilon }} \le 1$, where *σ* is the true flow stress and *ε* is the true strain. It is therefore obvious that ${\mathrm{\Theta }}$ (as seen in Fig. 5) has to stay high enough to keep up with the increasing stress *σ* for averting strain localization instability, so as to stabilize the uniform tensile plastic deformation before necking sets in at ${\mathrm{\Theta }}$=1. For any metal after strengthening via cold working or grain refinement, the slope of the stress–strain curve in the plastic flow regime is lower than for unstrengthened coarse-grained metals^7–10^, as the rate of defect accumulation becomes lower. This diminishes an effective strain-hardening mechanism in metals, i.e., the continuous multiplication and storage of dislocations during plastic straining. Consequently, after yielding at high stresses ${\mathrm{\Theta }}$ typically plunges towards unity quickly, such that an increased *σ*_y_ corresponds to a fast drop of *ε*_u_.

In this regard, the creation of heterogeneous nanostructures is particularly beneficial and can therefore be viewed as an overarching mechanism in promoting strength–ductility synergy. We make this point by first mentioning data point 11^21^ in Fig. 1. In^21^, the whole sample is just single-element Ni, but numerous nanograins misorientated with the Ni matrix serve the dual purpose of blocking and trapping dislocations. They are barriers to dislocations to increase strength, and simultaneously make dislocation motion sluggish to allow more dislocations to run into each other, react and multiply, elevating the storage rate of dislocations for strain hardening.

As we have discussed earlier, HEAs have a high propensity to develop heterogeneities, from subnanometer scale and up, in multiple forms and at various levels, above and beyond the case for elemental metal (Ni)^21^. First, because the alloy contains several species at high concentrations, statistically there is always a fluctuation of local chemical composition, even for a nominally random solution (Fig. 8). Each local region deviates from the global composition, leading to spatially varying SFE, dislocation core configurations, misfit volume, and distortions. Such inhomogeneities, especially when some degree of LCO is involved, lead to a rugged energy landscape more difficult for a traversing dislocation. This nanoscale trapping of dislocations^89^ presents short-distance obstacles to strengthen HEAs in a different way from conventional solid solution hardening, where each solute atom interacts separately with a dislocation through an elastic strain field.

Second, the multi-principal-element lattice often entails a low SFE. This may be perceived as follows: the “correct” stacking is already a complex one to begin with, so a faulted packing does not incur much additional energy penalty. The low SFE encourages the accumulation of stacking faults and twins (often nanoscale ones), during homogenization annealing as well as during plastic deformation. These accumulated defects are heterogeneously distributed and concentrated at grain corners, dynamically refining the grains. This elevates flow stress and contributes to strain hardening, as the defects are stored on the fly during tensile deformation. The next layer of heterogeneity comes from partial recrystallization that gives rise to different-sized grains mixed together. In the resultant HGS, soft regions deform plastically more than hard regions, so that gradients of plastic strain build up. Accommodation of such plastic gradients requires the storage of geometrically necessary dislocations^11^, which contribute to work hardening through a nonlocal effect of strengthening. Besides deformation twinning, phase transformation via TRIP gives rise to HCP and/or tetragonal-structured martensites. Last but not least, one can intentionally design microstructures composed of desired phases intermixed on nanoscale; e.g., an FCC ductile HEA matrix can suppress the brittleness tendency of precipitated phases^26^. All of these heterogeneities contribute effectively to strengthening and work hardening. Figure 5 displays a few examples of recent HEAs/MEAs (some are in Fig. 2) that showed sustainable strain-hardening rate to delay plastic instability, thus prolonging *ε*_u_.

### Discussion and concluding remarks

Multicomponent “high-entropy” alloys have become a new and fertile playground to metallurgists. The general rationale that has stimulated widespread interest in these HEAs is that unprecedented properties may emerge from the vast compositional space previously inaccessible. In the arena of strength–ductility synergy, this is also the case. We have begun to see strength–ductility combinations beyond current benchmark ranges, as shown in Fig. 1.

Our message is that, compared with simple metals and traditional solutions^94^, the concentrated HEAs are more conducive to heterogeneous microstructure and hence tend to be plastically nonhomogeneous^95^. Pronounced strengthening naturally follows from these roadblocks to dislocations, elevating the back stresses to unusually high levels (see Fig. 4), and work hardening is more proficient as well due to the increased likelihood for dislocations to stall, cross-slip^22,96^, interact, multiply, and accumulate. This sustains a strain-hardening rate that rivals or even exceeds that of unstrengthened metals (see Fig. 5), delaying plastic instability and prolonging a *ε*_u_ previously uncommon for high-strength metals. Here we recast the contributing factors to strengthening and strain hardening of these alloys in a different light. First, defect storage in HEAs is efficient, especially if the alloy has a low SFE, as shown for the HGS MEA, where deformation twins and faults are dynamically embedded to increase the defect content and refine the grains. Second, statistical composition variations and even LCO or size-misfit induced inhomogeneities are inevitable, sometimes enhanced by the addition of a substituting alloying element such as the case of Pd in the Cantor alloy^96^, or a small percentage of solutes such as oxygen, as shown in the case of (O,Ti,Zr)-complexes in TiZrNbHf BCC-based HEA^22^. Third, a second phase can be mixed in (as closely spaced nanoparticles), adding obstacles to cause strengthening and strain hardening. Fourth, complex HEAs make it possible to tune the composition (and hence SFE) such that twins and martensites can develop readily and store dynamically during plastic deformation^97^. TRIP effects akin to those in steels have been brought into the picture in many HEAs, adding interphase slip as yet another layer of difficulty against dislocation motion. In other words, HEAs contain many more heterogeneities than the structural ones available in elemental metals in Fig. 1. Note that it is the complexity and versatility of multicomponent alloys that allow the simultaneous superposition of several levels of heterogeneities, which in turn effectively promotes strength–ductility synergy.

While it has been repeatedly claimed in the literature that the strength–ductility trade-off can be overcome in some microstructures, these simultaneously increased *σ*_y_ and *ε*_u_ were observed with reference to a strengthened alloy that has already compromised ductility^22,33^. However, there are now indeed a few cases in Figs. 1 and  3, where the alloy has *σ*_y_ on GPa level, while its ductility remains as high as *ε*_u_ = 50%, even exceeding or at least equaling to that of an unstrengthened metal^26^. This defies the general trade-off trend and truly breaks the strength–ductility paradox.

Before closing, we mention in passing that at cryogenic temperatures (such as liquid nitrogen temperature) a remarkable strength–ductility synergy can be achieved with FCC HEAs^32,51,90,96,97,98^. FCC metals and alloys have long been known to simultaneously show higher strength and increased ductility at cryogenic deformation temperatures: in textbooks, there is no strength–ductility trade-off for FCC metals when temperature is lowered from RT to 77 K (and even to 4 K). This is because dynamic recovery diminishes at cryogenic temperatures such that dislocation accumulation is more effective to elevate strain-hardening rate and *ε*_u_. FCC HEAs/MEAs are particularly conducive to high strain-hardening rate at cryogenic temperatures, as exemplified by CrCoNi^32,98^: at 77 and 4 K the deformation stress is high and the already-low SFE is further reduced, both favoring pronounced deformation twinning and defect storage. It is therefore not surprising that this MEA’s strain-hardening rate and *ε*_u_ at 77 K are as impressive as the best 316 austenitic stainless steels. These alloys are well suited for cryogenic applications due to such a desirable low-temperature strength–ductility synergy.

We envision that the new multi-principal-element paradigm will continue to expand the repertoire of alloys not only in their compositions but also in their (*σ*_y_, *ε*_u_) properties. A high density of heterogeneities in the microstructure has become easy to come by, is often dynamically reinforced during tensile deformation, and builds a hierarchy consisting of spatial variations, planar defects, clusters/precipitates, grain-size distribution, and second phases. This has been illustrated using recent HEA examples, including single-phase solution HEAs/MEAs, under a common umbrella of heterogeneities either enabled or facilitated by multiple principal elements. We project continued endeavors towards refined and enhanced HEAs to achieve superior properties. In particular, opportunities abound to tailor the heterogeneities to ward off plastic instabilities and realize even better strength–ductility balance.
